# Inhibiting PHD2 in bone marrow mesenchymal stem cells via lentiviral vector-mediated RNA interference facilitates the repair of periodontal tissue defects in SD rats

**DOI:** 10.18632/oncotarget.20243

**Published:** 2017-08-14

**Authors:** Changxing Chen, Houxuan Li, Jun Jiang, Qian Zhang, Fuhua Yan

**Affiliations:** ^1^ Nanjing Stomatological Hospital, Medical School of Nanjing University, Nanjing, Jiangsu, 210008, China; ^2^ Central Laboratory of Stomatology, Nanjing Stomatological Hospital, Medical School of Nanjing University, Nanjing, Jiangsu, 210008, China; ^3^ Stomatological Hospital of Fujian Medical University, Fuzhou, Fujian, 350001, China

**Keywords:** lentiviral vector, PHD2, stem cells, periodontal tissue defects, oxidative stress

## Abstract

Hypoxia-inducible factors (HIFs) play an important role in angiogenesis, and they can activate the expression of several downstream angiogenic factors. HIF-1 is a major transcriptor of HIFs, composed of α and β subunits. Prolyl hydroxylase domain-containing protein 2 (PHD2) is the main catabolic enzyme for HIF-1α, and it can accelerate its degradation under normoxic conditions. PHD2 expression in bone marrow mesenchymal stem cells (BMMSCs) of SD rats was down-regulated under normoxic conditions in this study by utilizing lentiviral vector-mediated RNA interference to promote HIF-1α accumulation, thus enhancing the expression of angiogenic factors. A tissue-engineered compound was constructed using the composite collagen membrane of BMMSCs after PHD2 gene silencing to repair periodontal fenestration defects in SD rats. The results of this study indicated that, after PHD2 gene silencing, the osteogenic differentiation of BMMSCs was enhanced *in vitro*, the resistance of cells to oxidative stress was also validated *in vitro*, thereby illustrating the promotion of the repair of artificially constructed periodontal tissue defects in rats. The results of this study provide a reference and guidance for future applications of RNA interference in periodontal tissue engineering and serve as a basis for improving the survival of seed cells in recipient tissues.

## INTRODUCTION

Periodontitis is one of the most common chronic oral diseases. It is an clinical condition that must be urgently treated as it can lead to periodontal tissue detachment and alveolar bone defects [1, 2]. Rapidly developing tissue engineering and gene technology in recent years has allowed many investigators to use seed cells that are favorable for tissue reconstruction, especially to construct tissue scaffolds for use in regenerative therapy for periodontal tissue defects [3, 4]. Bone marrow mesenchymal stem cell (BMMSCs) with multi-potent cells can be used, as they differentiate into several tissue and cell types under different conditions, e.g., fat, bone, cartilage, muscle, etc. BMMSCs also have inducing effects on hematopoietic stem cells and are considered to be the ideal seed cells for tissue repair in tissue engineering [5].

Based on several in-depth studies on tissue engineering technology, many scholars believe that more seed cells are required for larger and more complicated tissue defect repairs using this technology, and extensive vasculature is needed to provide seed cells with adequate nutrients [6, 7]. Achieving bone regeneration and revascularization for periodontal tissues and creating a suitable environment for the normal growth of hard and soft tissues required for the restoration of normal structure and functions have become critical issues in the field of periodontal tissue engineering.

Several studies in recent years have confirmed that hypoxia-inducible factors (HIFs) play a crucial role in revascularization [8, 9]. HIF-1 is a major transcriptor for HIFs and is a heterodimer composed of α and β subunits. HIF-1α unit is favorable for binding to its corresponding HIF-1β unit to form a heterodimer structure, thus realizing the effect of HIF-1 as a major transcription factor [10]. HIF-1β is stably expressed in the cytoplasm. Prolyl hydroxylase (PHD) can hydroxylate proline residues in the oxygen-dependent degradation domain (ODDD) of HIF-1α subunits by VHL E3 ubiquitination under normoxic conditions, thus promoting HIF-1α degradation by the ubiquitin-proteasome system. PHD is inhibited under hypoxic conditions, as it utilizes oxygen as its auxiliary substrate, which can inhibit HIF-1α degradation. HIF-1α and HIF-1β combine to form active HIF-1 under hypoxic conditions, thus promoting nuclear transcription of several downstream genes [11, 12]. HIF-1 activates more than 70 species of downstream genes and proteins primarily involved in red blood cell production, angiogenesis, cytokine production, energy metabolism, cell survival and apoptosis, and other physiological and pathological processes [13]. The angiogenic factors regulated by HIF-1 include vascular endothelial growth factor (VEGF), erythropoietin (EPO), inducible nitric oxide synthase (INOS) and basic fibroblast growth factor (bFGF), thereby promoting revascularization and improving the function of ischemic tissues [14]. It has been found that prolyl hydroxylase domain protein 2 (PHD2) activity is stronger in prolyl hydroxylases and is believed to be the dominant catabolic enzyme for HIF-1α [15].

Periodontal tissue defects that result from periodontitis can remain as chronic sources of infection despite early treatment of periodontal disease. It has been shown that an imbalance exists in concentrations of reactive oxygen species (ROS) and antioxidant in periodontitis. Excessively high ROS concentrations result in a series of toxic stimulations to periodontal tissues and cells, inducing apoptosis, and even activating and inducing osteoclasts, which subsequently result in the destruction of periodontal support tissues, causing oxidative stress [16, 17]. Therefore, when a tissue-engineered compound is implanted into a periodontal defect, it is critical to increase the resistance of the seed cells to oxidative stress, which enhances seed cell survival and effectively enhances tissue repair by the tissue-engineered compound.

In recent years, gene therapy has evolved, and RNA interference is extensively being applied in the field of tissue engineering. Due to the desirable results of RNA interference in tissue regeneration, some scholars believe that RNA interference is one of the important measures for periodontal tissue regeneration [18]. Viral gene vectors have been extensively used in previous studies. Lentiviral vectors are known to extensively infect cells and can integrate target genes into the genome of target cells, which can then have long-term stable expression. In recent years, several studies have been performed to successfully achieve RNA interference of target genes through lentiviral vectors [19, 20].

Hence, in this study, under normoxic conditions, PHD2 expression in BMMSCs of Sprague Dawley (SD) rats was down-regulated by utilizing lentiviral vector-mediated RNA interference to realize gene silencing and to promote HIF-1α accumulation as well as the action of angiogenic factors, including downstream VEGF. A tissue-engineered compound construct using the composite collagen membrane of BMMSCs with PHD2 gene silencing was implanted in a rat periodontal fenestration defect model. The osteogenic differentiating capacity of BMMSCs and the enhanced effects of angiogenic factors on angiogenesis and bone regeneration were utilized to promote repair and reconstruction of the periodontal tissue defects. *In vitro* oxidative stress was simultaneously induced to validate the resistance of BMMSCs to oxidative stress after PHD2 gene silencing, thereby serving as a new basis for improving seed cell survival in periodontal tissue engineering.

## RESULTS

### Cultivation of BMMSCs

**Figure 1:**
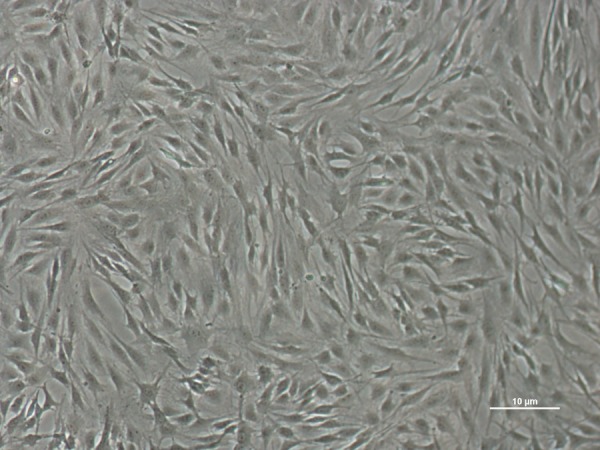
Growth of third generation BMMSCs from SD rats (×100)

The recovered cells were passaged to the third generation, and microscopic findings suggested that the BMMSCs were primarily comprised of spindle cells with a few polygonal and stellate cells. Cells grew well and were arranged in radial or spiral shapes with cell colonies, and more than 80% of the cells were fused (Figure 1). Third generation BMMSCs were used for subsequent experiments.

### Lentiviral vector infection of BMMSCs at different MOI values

**Figure 2:**
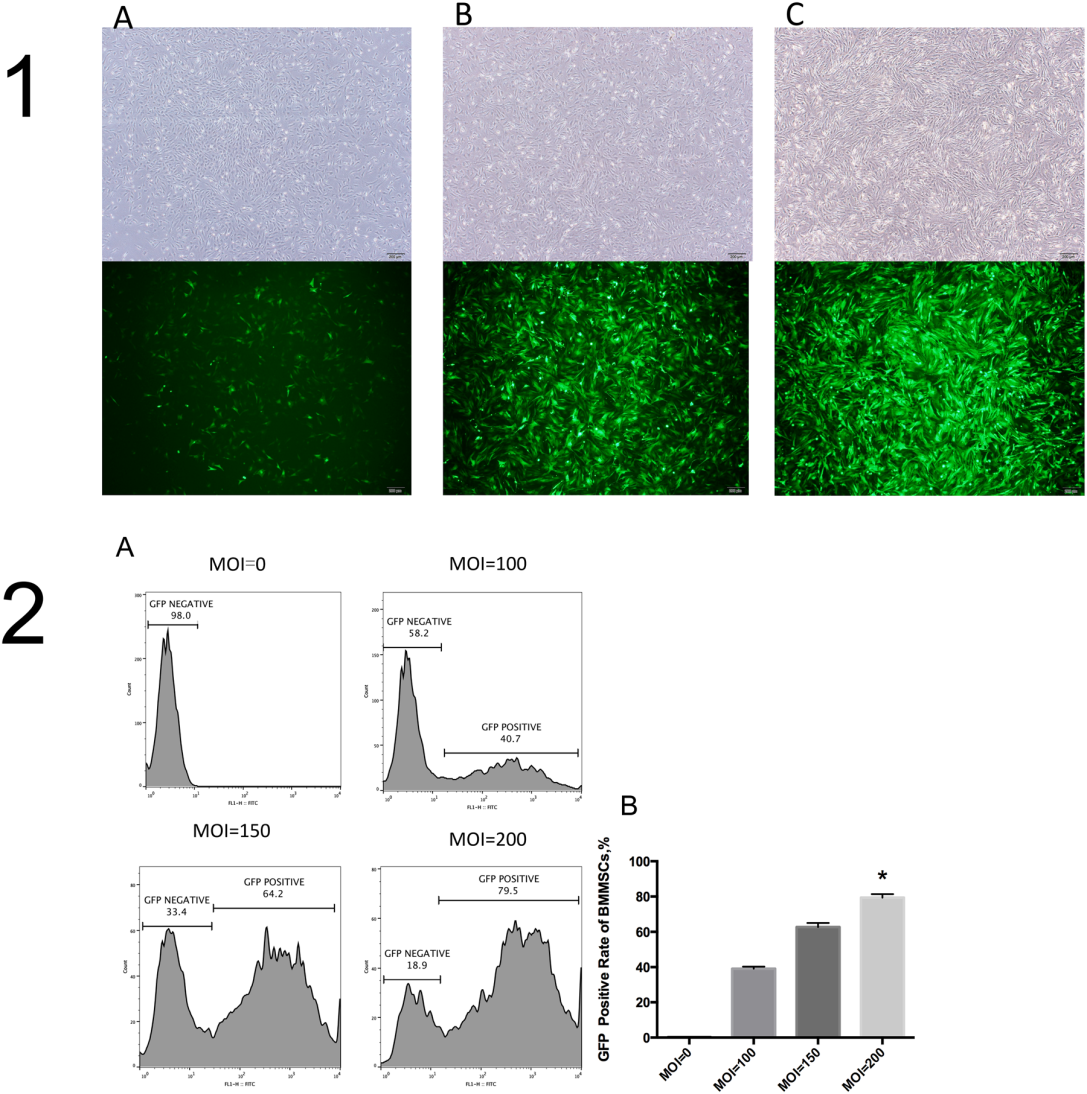
BMMSCs at different MOI values **(1)** Bright field images of cells and images under fluorescence microscope at different MOI values 72 hours after infection (×40). **(1A)**, MOI=100; **(B)**, MOI=150; **(C)**, MOI=200. **(2A)** Cell flow charts at different MOI values; **(2B)** GFP positive expression rate at different MOI values in 1B different groups, 1C * *vs*. other groups, *P*<0.05.

When viewed under a bright field microscope 72 hours after infection, BMMSCs grew well after infection in different groups. No obvious differences were observed. Cells were primarily long, spindle-shaped and striped, arranged in radial or vortex shapes, and partially fused. Under an inverted fluorescence microscope, an obviously different intensity of protein expression was observed in green florescence. The MOI200 group was observed to have a significantly higher intensity of green florescent protein expression than the MOI150 and MOI100 groups (Figure 2-1).

Flow analysis results indicated that GFP positive expression rate was nearly 80% in the MOI200 group, significantly higher than those in the MOI150, MOI100 and MOI0 groups (P<0.05)(Figure 2-2), respectively. It was believed that at MOI200, BMMSC lentiviral vector infection rate reached 80%, which was an effective concentration of infection.

### Target genes and protein expression after BMMSCs were infected with PHD2 gene lentivirus RNA interference vectors

**Figure 3:**
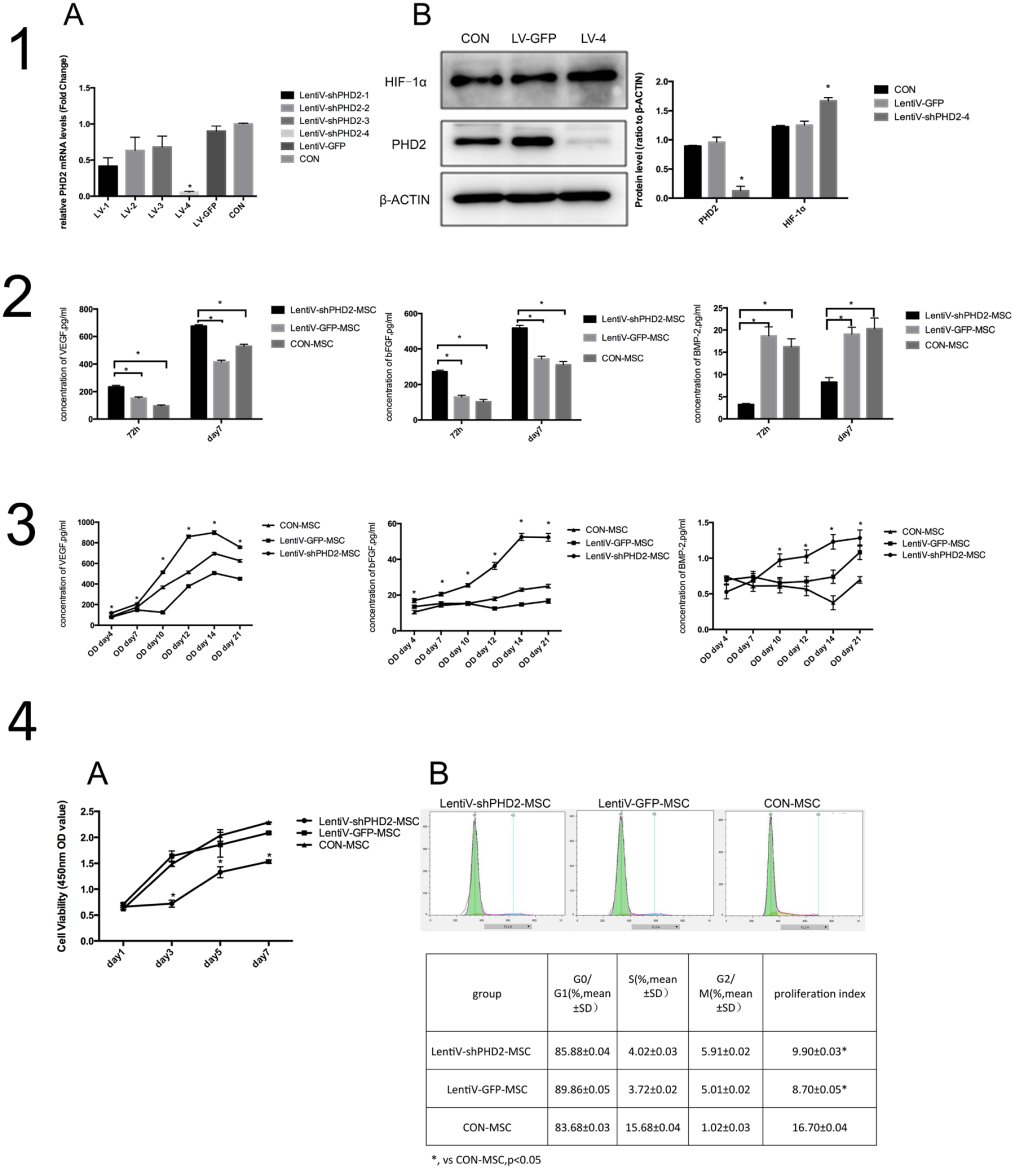
Biological behaviors of BMMSCs after PHD2 gene silencing *in vitro* **(1A)** PHD2 mRNA expression level in different groups 72 hours after lentiviral vector infection, * *vs*. other groups, *P*<0.05. **(1B)** expression levels of PHD2 and HIF-1α in the LentiV-shPHD2-4 group 72 hours after lentiviral vector infection, * *vs*. other groups, *P*<0.05. **(2)** Concentrations of VEGF, bFGF and BMP-2 in the supernatants from each group 72 hours and 7 days after infection; *, *P*<0.05. **(3)** From days 4 to 21 of osteogenic induction, the concentrations of VEGF (A), bFGF (B) and BMP-2 (C) in the supernatants in the different groups at different time points, *, *vs*. other groups, *P*<0.05. **(4A)** Cell proliferation in the lentiV-shPHD2-MSC, lentiV-GFP-MSC and CON-MSC groups within 1 week. *, *vs*. other groups, *P*<0.05; **(4B)** Detection results of cell cycles in the lentiV-shPHD2-MSC, lentiV-GFP-MSC and CON-MSC groups.

Cells in different groups were infected for 72 hours by a lentiviral vector. A real-time quantitative PCR showed that the relative expression levels of PHD2 mRNA in the LentiV-shPHD2-4 group was significantly lower than those in the other groups (P<0.05) (Figure 3-1A). The total protein was detected by Western blot in the Lenti-shPHD2-4 group, the Lenti-GFP group (negative control group) and the blank control group (non-infection group). The results showed that, after 72 hours of exposure to infection, the expression level of PHD2 total protein in the LentiV-shPHD2-4 group was significantly lower compared to the other groups, and the expression level of downstream HIF-1α protein was significantly increased (P<0.05) (Figure 3-1B). The above results indicated that a constructed lentiviral RNA interference vector (LentiV-shPHD2-4) could silence the BMMSC PHD2 gene under normoxic conditions and activate pathways related to downstream HIF-1, making it favorable for subsequent studies.

### PHD2 gene silencing and its effects on the biological behavior of BMMSCs *in vitro*

After exposure to lentiviral infection for 72 hours and 7 days, respectively, VEGF and bFGF concentrations in the supernatant from the lentiV-shPHD2-MSC group were significantly higher than in the negative control and blank control groups (P<0.05). BMP2 expression in the supernatant from the lentiV-shPHD2 group was lower than in the negative control and blank control groups (P<0.05). Compared to day 3 (72 h), the concentrations of growth factors increased in each group on day 7 (Figure 3-2). The results indicated that, during the early phase, PHD2 gene silencing can promote the secretion of VEGF and bFGF in BMMSCs under the normal state but inhibits the secretion of osteogenic-associated protein BMP2.

From day 4 to 21 of osteogenic induction, the concentrations of vascular endothelial growth factor (VEGF)and basic fibroblast growth factor(bFGF) in the groups gradually increased; they peaked on day 14 and decreased on day 21. On days 4, 7, 10, 12, 14 and 21, the concentrations of VEGF and bFGF in the supernatant were all higher in the lentiV-shPHD2-MSC group than in the LentiV-GFP-MSC and CON-MSC groups (P<0.05). The results indicated that PHD2 gene silencing could promote the secretion of angiogenic factors in BMMSCs in early, mid- and late phases. Bone morphogenic protein2(BMP2)concentration in the supernatant of the lentiV-shPHD2-MSC group generally tended to increase from days 4 to 21 and peaked on day 21, but no significant difference was observed in BMP2 concentration in the supernatant during the first week of osteogenic induction in the LentiV-GFP-MSC and CON-MSC groups. From days 10 to 21, the BMP2 concentration in the supernatant from the lentiV-shPHD2-MSC group persistently increased and was higher than the concentrations in the LentiV-GFP-MSC and CON-MSC groups during the same period (P<0.05). The results indicated that PHD2 gene silencing could promote BMP2 secretion in the mid- and late phases of BMMSC osteogenic induction. However, such effects were not obvious in the early osteogenic induction phase (Figure 3-3).

**Figure 4:**
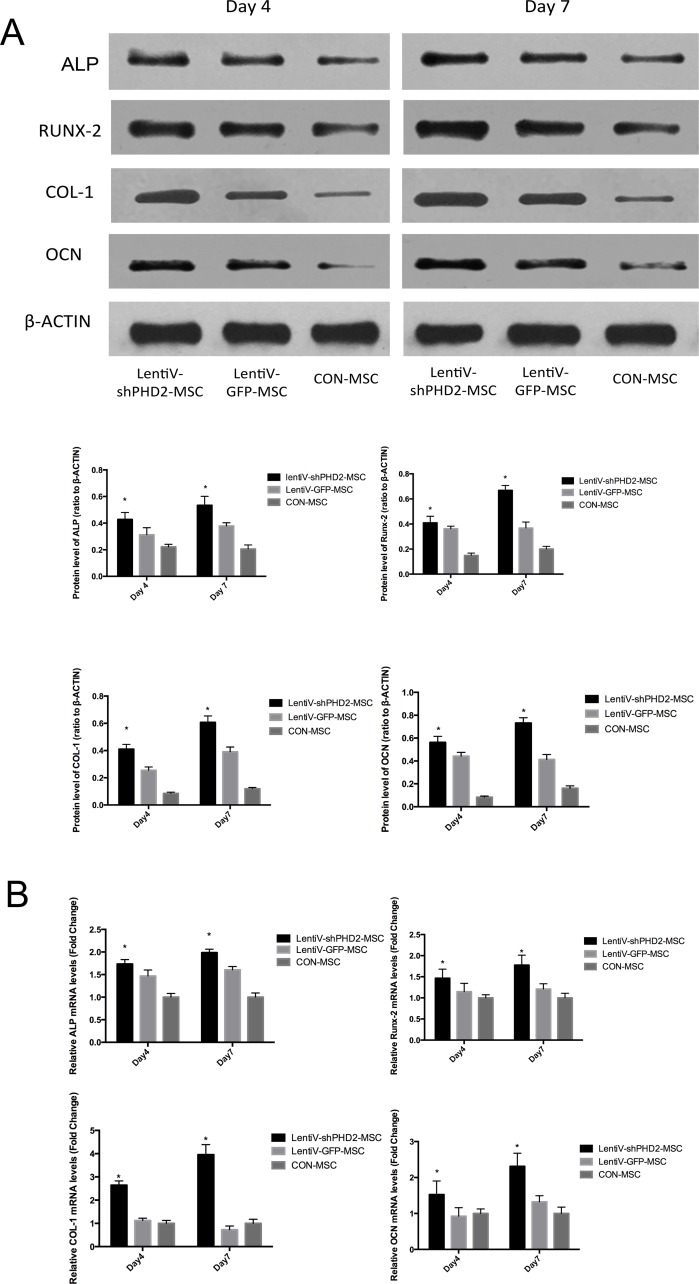
Expression levels of osteogenesis-related parameters during the early osteogenic induction **(A)** On days 4 and 7 of osteogenic induction, total protein levels of osteogenesis-related parameters including Runx-2, ALP, OCN and COL-1 in the different groups; **(B)** On days 4 and 7 of osteogenic induction, mRNA expression levels of osteogenesis-related parameters including Runx-2, ALP, OCN and COL-1 in the different groups. *, *vs*. other groups, *P*<0.05.

The cell viability of the lentiV-shPHD2-MSC group on day 1, 3, 5 and 7 after implantation were lower than those in the lentiV-GFP-MSC and CON-MSC groups (P<0.05). The cell proliferation curve tended to ascend within 1 week in the different groups (Figure 3-4A). The results indicated that PHD2 gene silencing had certain inhibitory effects on the proliferation rate of BMMSCs after 1 week. After lentiviral infection for 72 hours, the detection results of cell cycles in the lentiV-shPHD2-MSC, LentiV-GFP-MSC and CON-MSC groups showed that the cell proliferation indices of the lentiV-shPHD2-MSC and LentiV-GFP-MSC groups were lower than that of the CON-MSC group (P<0.05) (Figure 3-4B). The results indicated that lentiviral vector infection could affect cell proliferation in early phases.

On days 4 and 7 of osteogenic induction, total protein and mRNA expression levels of osteogenesis-related parameters including Runt-related transcription factor 2 (Runx-2), alkaline phosphatase (ALP), osteocalcin (OCN) and Collagen Type I (COL-1) in the lentiV-shPHD2-MSC groups were higher than those in the LentiV-GFP-MSC and CON-MSC groups (P<0.05). The results indicated that PHD2 gene silencing could promote early osteogenic differentiation of BMMSCs (Figure 4).

**Figure 5:**
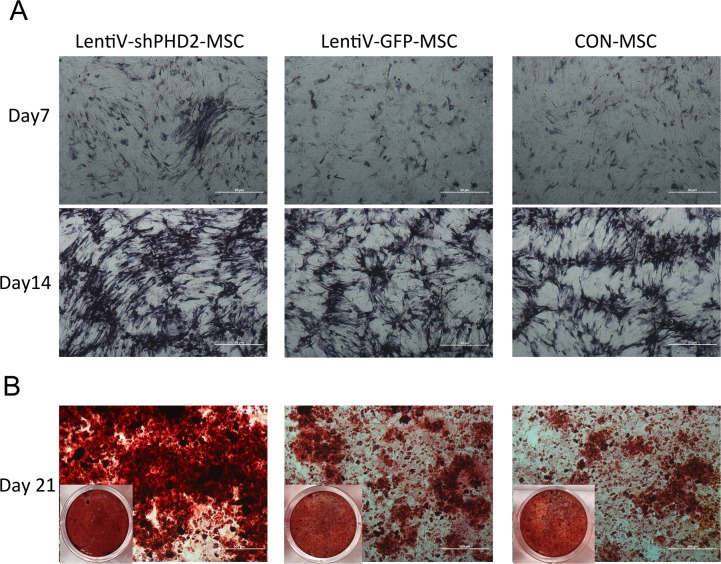
Alkaline phosphatase and alizarin red staining during the osteogenic induction **(A)** ALP-specific staining results on days 7 and 14 of osteogenic induction (×40); **(B)** Alizarin red staining results on day 21 of osteogenic induction (×40).

On days 7 and 14 of osteogenic induction, the ALP-specific staining results showed that, compared to the LentiV-GFP-MSC and CON-MSC groups, the lentiV-shPHD2-MSC group exhibited deeper staining, suggesting that ALP activity was higher in the PHD2 gene-silenced BMMSCs during osteogenic induction. On day 21 of osteogenic induction, alizarin red staining results showed that, compared to the LentiV-GFP-MSC and CON-MSC groups, the lentiV-shPHD2-MSC group showed more mineralized nodules, deeper staining, and larger staining area, suggesting that PHD2 gene silencing could promote osteoblast mineralization of BMMSCs *in vitro* (Figure 5).

### PHD2 gene silencing can enhance BMMSC resistance to oxidative stress

**Figure 6:**
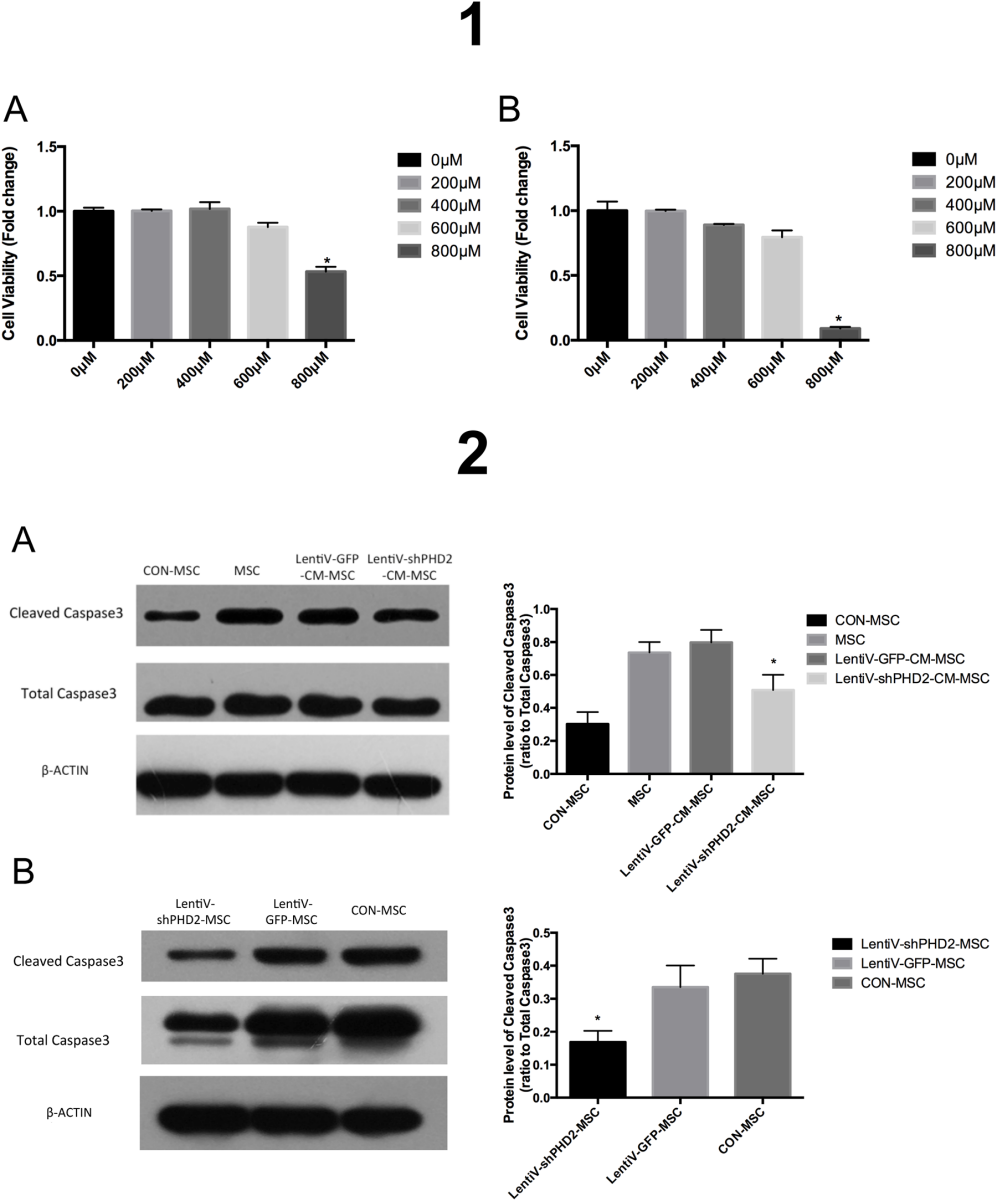
Cell viabilities in different concentration of H_2_O_2_ and Expression levels of the apoptosis proteins **(1A)** Cell viabilities in different groups after BMMSCs were treated with different concentration gradients of H_2_O_2_ for 3 hours; **(1B)** Cell viabilities in different groups after BMMSCs were treated with different concentration gradients of H_2_O_2_ for 6 hours. *, *vs*. other groups, *P*<0.05. **(2A)** Expression levels of cleaved Caspase-3/total Caspase-3 proteins after 800 μM H_2_O_2_ treatment for 6 hours in the CON-MSC, LentiV-shPHD2-CM-MSC, LentiV-GFP-CM-MSC and MSC groups; **(2B)** Expression levels of cleaved Caspase-3/total Caspase-3 proteins after 800 μM H_2_O_2_ treatment for 6 hours in the LentiV-shPHD2-MSC, LentiV-GFP-MSC and CON-MSC groups. *, vs. other groups, P<0.05.

After BMMSCs were treated with different concentration gradients of H_2_O_2_ for 3 or 6 hours, the results indicated that, at 800 μM, cell viability was significantly reduced compared to other concentration gradients (P<0.05) (Figure 6-1). Therefore, 800 μM H_2_O_2_ was selected as the optimum condition to imitate oxidative stress *in vitro* in subsequent experiments.

According to the Western blot results after the cell treatments described in section 6.2 of the Materials and Methods, when cells were treated with 800 μM H_2_O_2_ for 6 hours, cleaved Caspase-3 protein expression significantly decreased in the LentiV-shPHD2-CM-MSC group compared to the LentiV-GFP-CM-MSC and MSC groups (P<0.05). The total protein expression levels in cleaved Caspase-3 was still significantly different when comparing the LentiV-shPHD2-CM-MSC and control groups (CON-MSC group without H_2_O_2_ treatment) (P<0.05) (Figure 6-2A).

The Western blot results after cell treatment described in section 6.3 of the Materials and Methods showed that cleaved Caspase-3 protein expression was significantly decreased in the LentiV-shPHD2-MSC group compared to the LentiV-GFP- MSC and CON-MSC groups (P<0.05) (Figure 6-2B).

**Figure 7:**
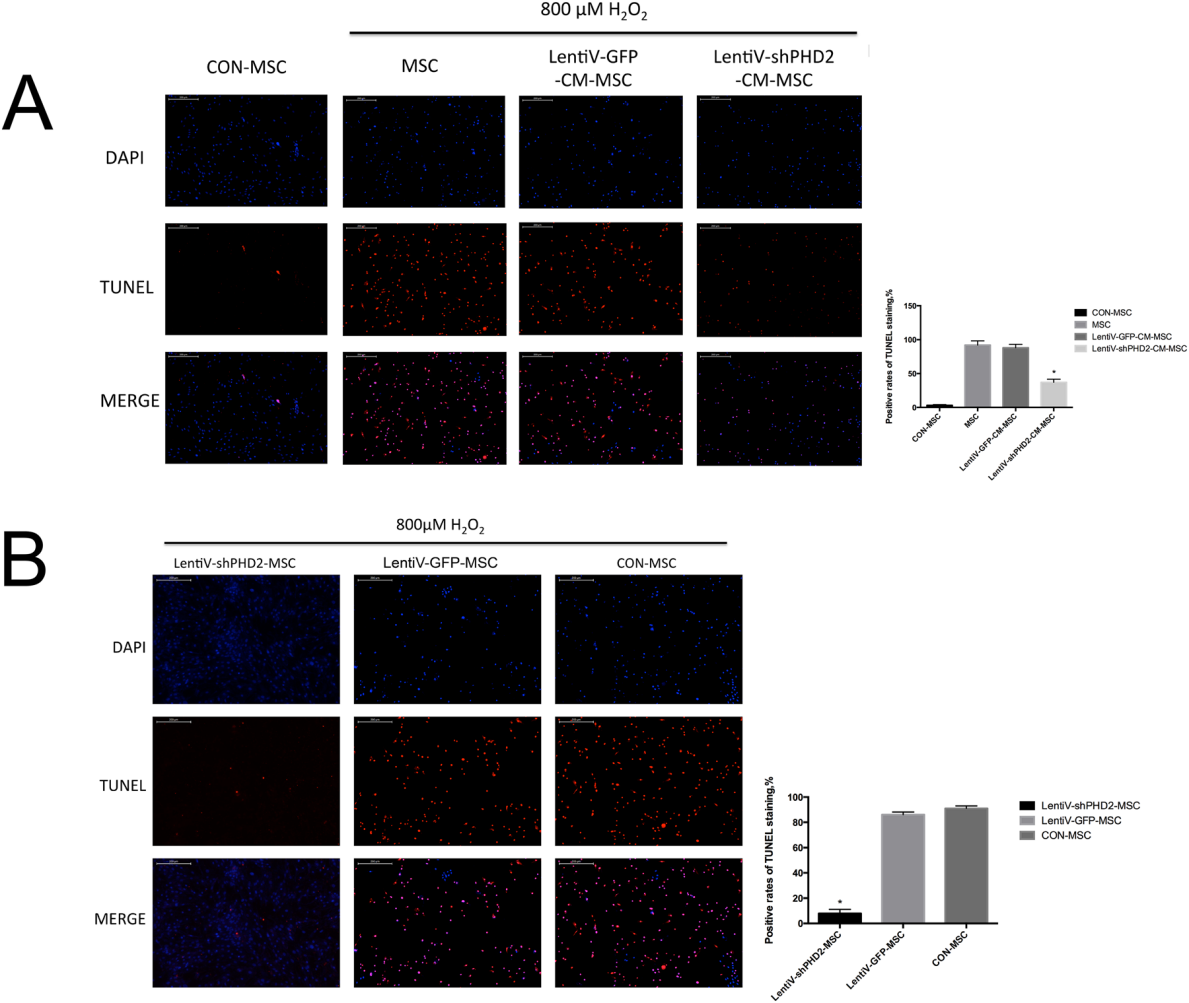
The images of the TUNEL stainings **(A)** DAPI staining, TUNEL staining and MERGE image in CON-MSC, LentiV-shPHD2-CM-MSC, LentiV-GFP-CM-MSC and MSC groups after 800 μM H_2_O_2_ treatment for 3 hours (×40) and the positive rates of TUNEL staining in the 3 groups. *, vs. other groups, *P*<0.05; **(B)** DAPI staining, TUNEL staining and MERGE image in the LentiV-shPHD2-MSC, LentiV-GFP-MSC and CON-MSC groups after 800 μM H_2_O_2_ treatment for 3 hours (×40) and the positive rates of TUNEL staining in the 4 groups. *, vs. other groups, *P*<0.05.

The cell growth TUNEL staining results described in sections 6.2 and 6.3 of the Materials and Methods were observed by fluorescence confocal microscopy. The results indicated that, after cells were treated with 800 μM H_2_O_2_ for 3 hours, positive TUNEL staining was significantly reduced in the LentiV-shPHD2-CM-MSC group compared to those in the LentiV-GFP-CM-MSC and MSC groups (P<0.05) but remained higher than that in the control group (CON-MSC group, no H_2_O_2_ treatment). Positive TUNEL staining significantly decreased in the LentiV-shPHD2-MSC group compared to in the LentiV-GFP-MSC and CON-MSC groups (P<0.05) (Figures 7A and 7B).

**Figure 8:**
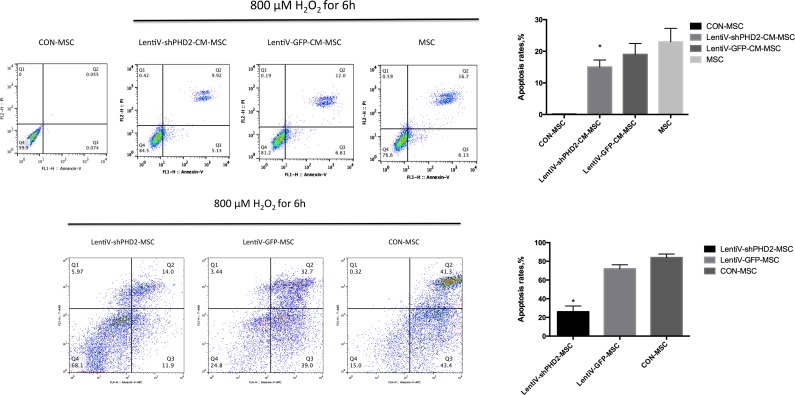
Flow cytometry of the apoptosis rates **(A)** FITC Annexin V/PI double staining flow charts and apoptosis rates in THE CON-MSC, LentiV-shPHD2-CM-MSC, LentiV-GFP-CM-MSC and MSC groups after 800 μM H2O2 treatment for 6 hours; **(B)** APC Annexin V/7-AAD double staining flow charts and apoptosis rates in THE LentiV-shPHD2-MSC, LentiV-GFP-MSC and CON-MSC groups after 800 μM H_2_O_2_ treatment for 6 hours. *, vs. other groups, *P*<0.05.

Flow cytometry results indicated that, after 800 μM H_2_O_2_ treatment for 6 hours, the apoptosis rate in the LentiV-shPHD2-CM-MSC group (early + late apoptosis rate) was lower than that in the LentiV-GFP-CM-MSC and MSC groups, and the apoptosis rates of these three groups were higher than in the untreated cells in the CON-MSC group (*P*<0.05). The apoptosis rate in the LentiV-shPHD2-MSC group was lower than those in the LentiV-GFP-MSC and CON-MSC groups (*P*<0.05) (Figure 8).

The above experimental results confirmed that the supernatant from cell culture after PHD2 gene silencing could protect BMMSCs and help cells resist H_2_O_2_-induced oxidative stress *in vitro*. PHD2 gene silencing could also enhance BMMSC resistance to H_2_O_2_-induced oxidative stress *in vitro*.

### BMMSCs after PHD2 gene silencing can promote local neovascularization *in vivo*

**Figure 9:**
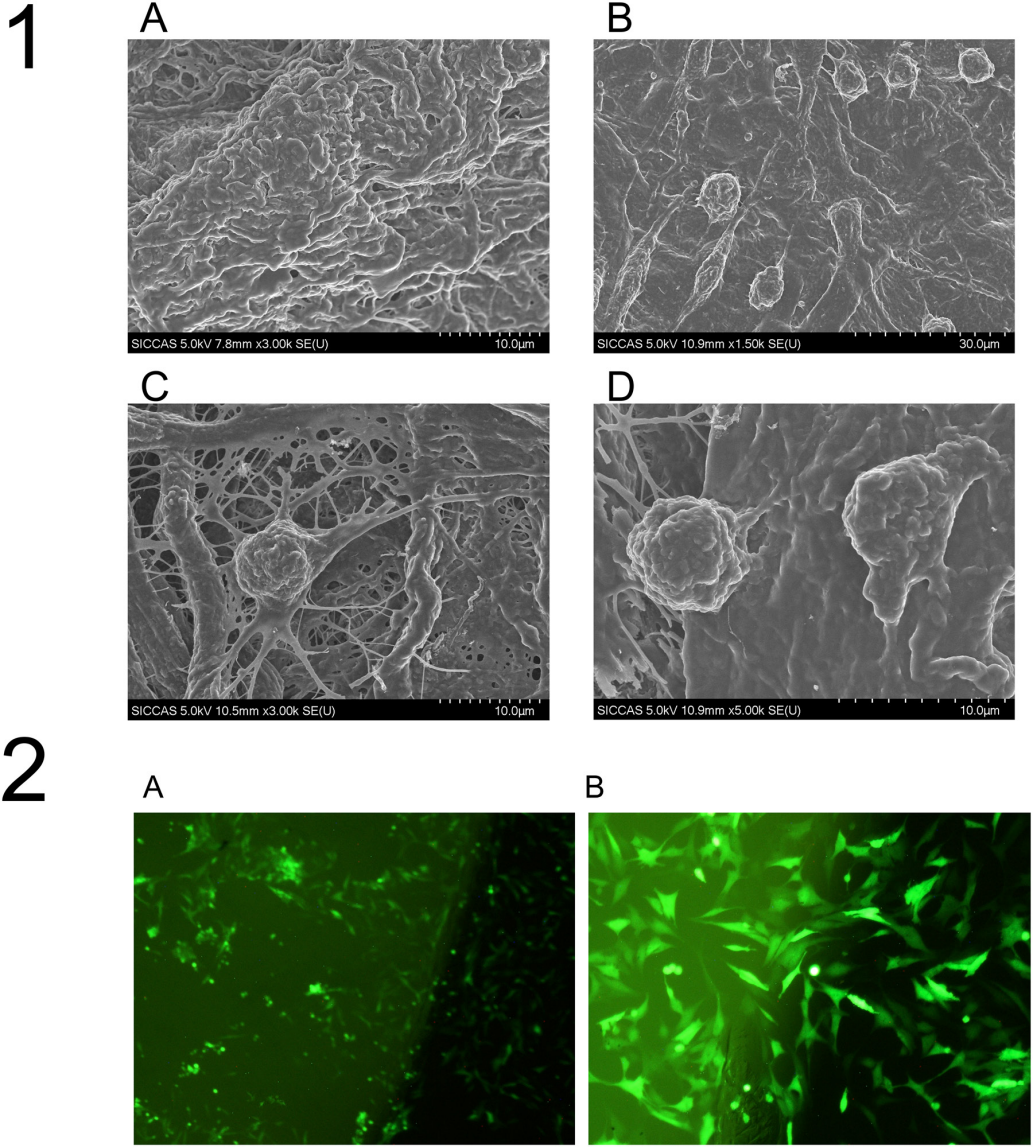
The surface structure of the tissue-engineered compounds by SEM **(1A)** shows the surface of the blank collagen membrane; **(1B)** **(1C)** and **(1D)** show the cells colonizing on the collagen membrane in the LentiV-shPHD2-MSC group at different magnifications (×1.5K, ×3K and ×5K). **(2)** Cell colonization on the collagen membrane in the LentiV-shPHD2-MSC group under fluorescence confocal microscopy **(2A)** ×40; **(2B)** ×100).

Scanning electron microscopy revealed that after PHD2 gene silencing, BMMSCs could successfully colonize the collagen membrane, with the cytoplasm extending evenly. The cell antennae could also deeply adhere to the internal fibers of the collagen membrane (Figure 9-1), suggesting that the tissue-engineered compound designed in this study was successfully constructed. Fluorescence confocal microscopy revealed that, when BMMSCs successfully colonized the collagen membrane after being infected, GFP was strongly expressed, and there was good cell growth (Figure 9-2).

**Figure 10:**
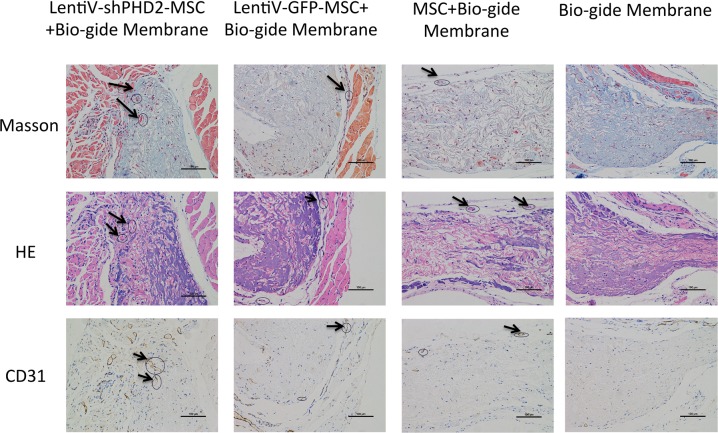
Histological observation of the tissue-engineered compound implanted subcutaneously in nude rats Images of histological sections after the tissue-engineered compound was subcutaneously implanted into nude rats (×40); black arrows indicate new blood vessels.

Findings from histological sections after the tissue-engineered compound was subcutaneously implanted in nude rats for 5 weeks indicate that the collagen membrane was not completely degraded in all groups; purplish red HE staining and MASSON's blue staining can be seen where collagen membranes had not been degraded. The internal collagen membrane fibers in the LentiV-GFP-MSC+Bio-Gide, CON-MSC+Bio-Gide and simple Bio-Gide groups had degraded more than in the LentiV-shPHD2-MSC+Bio-Gide group. Ample cell matrix and connective tissues had grown into the collagen membrane in each group. HE and MASSON staining showed neovascularization in the collagen membrane with large-diameter vessels in the LentiV-shPHD2-MSC+Bio-Gide collagen membrane group. Immunohistochemistry results were positive and showed CD31 as the vascular endothelial cell marker. In LentiV-GFP-MSC+Bio-Gide, CON-MSC+Bio-Gide and Bio-Gide collagen membrane groups, neovascularization was only observed in peripheral connective tissues or at the border between connective tissues and collagen membrane, where blood vessels had smaller diameters and were restricted in the local region (Figure 10).

### BMMSCs after PHD2 gene silencing may promote rat periodontal fenestration defect repair *in vivo*

#### Observation and analysis of histological sections

**Figure 11:**
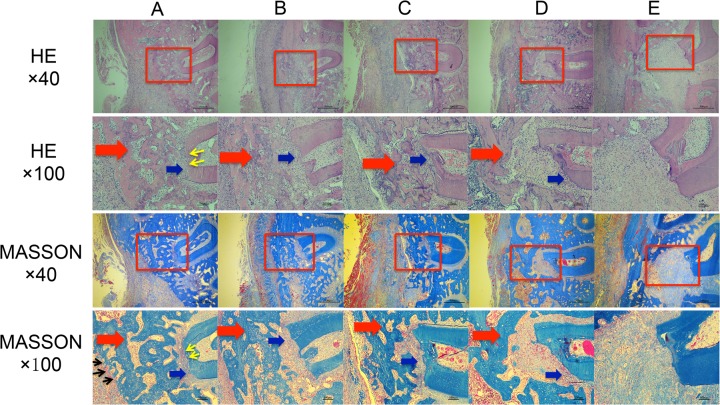
Histological observation of the repair of periodontal tissue defects in different groups **(A)** LentiV-shPHD2-MSC+Bio-Gide collagen membrane group; **(B)** LentiV-GFP-MSC+Bio-Gide collagen membrane group; **(C)** CON-MSC+Bio-Gide collagen membrane group; **(D)** simple Bio-Gide collagen membrane; **(E)** blank control group. Red arrow indicates NB, blue arrow indicates NP, black arrow indicates new blood vessel, yellow arrow indicates NC.

Specimen sections were stained via HE and MASSON methods 3 weeks after surgery and observed under a microscope (Figure 11). Varying degrees of neonatal alveolar bone (NB) repair and disordered loose structures of neonatal periodontal ligament (NP) repair were observed at the periodontal defect sites in all groups, except the E group. The E group corresponded to the blank control group, in which large areas of disordered loose fibrillar structures were observed at the bucco-distal root of the first molar, and no obvious NB were observed. Varying degrees of NB repair and disordered loose structures of NP repair were observed at the bucco-distal root of the first molar in the B, C, and D groups, which showed loose trabecular bone and obvious local bone resorption. Compared to the other 4 groups, group A (LentiV-shPHD2-MSC+Bio-Gide collagen membrane group) showed larger areas of NB at the bucco-distal root of the first molar with more prominent NP repair. The NB in group A showed a more compact structure than the other groups, and local neovascularization was observed near the NB. The neonatal cementum (NC) was also observed at the bucco-distal root of the first molar in group A, although the structure was not obvious. Histological findings at 3 weeks showed that group A (LentiV-shPHD2-MSC+Bio-Gide collagen membrane group) promoted more obvious periodontal tissue repair, including more NB, NP and NC, than the other groups during the early phase. These results were significant in the mature and complete periodontal tissue repair during the later phase.

**Figure 12:**
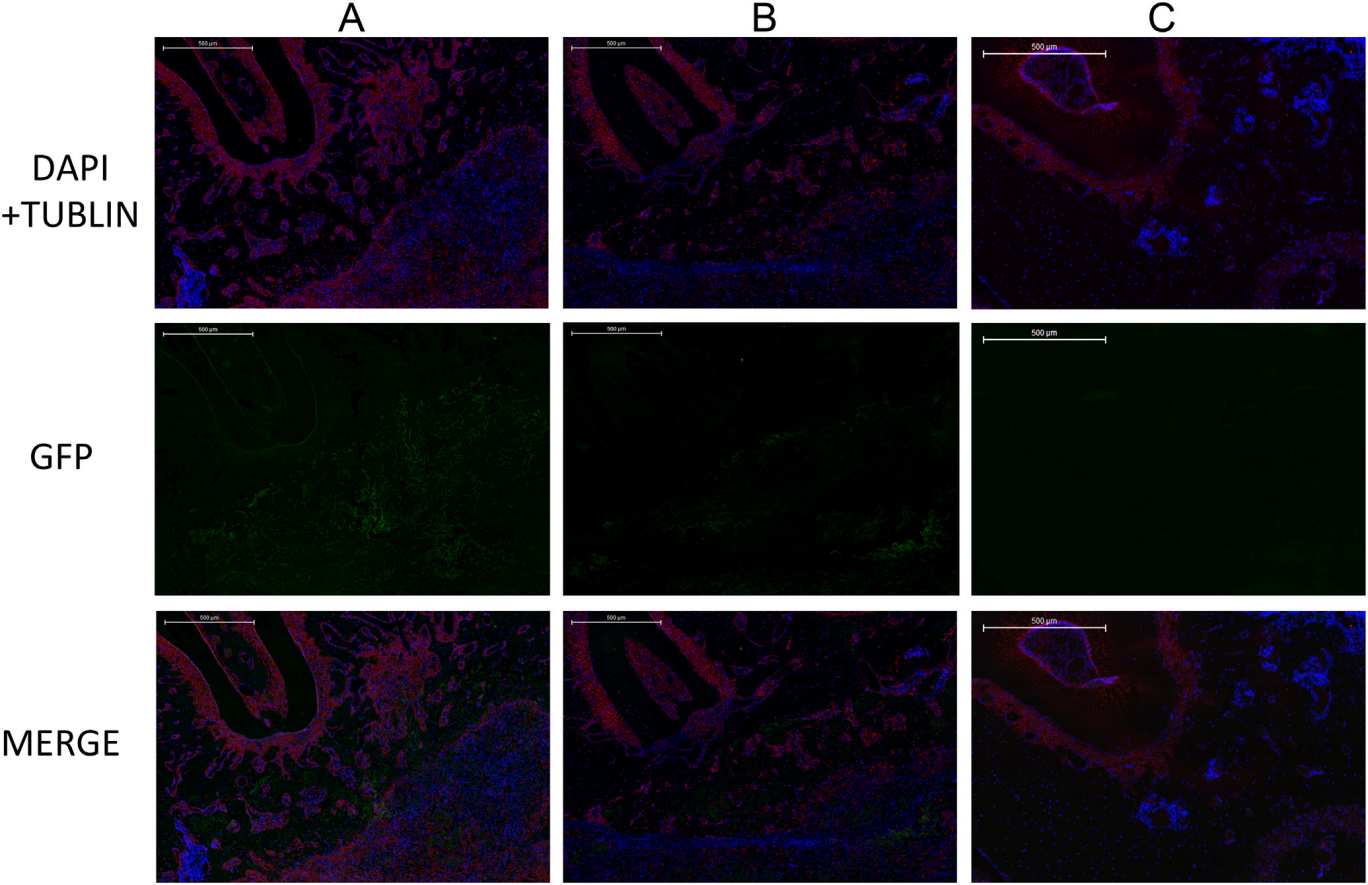
Immunofluorescence images of GFP-positive region in different groups **(A)** LentiV-shPHD2-MSC+Bio-Gide collagen membrane group; **(B)** LentiV-GFP-MSC+Bio-Gide collagen membrane group; **(C)** CON-MSC+Bio-Gide collagen membrane group(×50).

Immunofluorescence results showed that a GFP-positive region could be observed at the bucco-distal root of the first molar in both the LentiV-shPHD2-MSC+Bio-Gide collagen membrane group and the LentiV-GFP-MSC+Bio-Gide collagen membrane group. Based on the above results, we considered the GFP-positive region as the NB area. The CON-MSC+Bio-Gide collagen membrane group showed no significant GFP positive region(Figure 12). As GFP-labelled BMMSCs were implanted in both groups, we believed that the implanted seed cells (BMMSCs) began to undergo osteogenic differentiation and the NB formation process in this region. Larger GFP-positive regions were observed in the LentiV-shPHD2-MSC+Bio-Gide collagen membrane group. The above results showed that the constructed tissue-engineered compound promoted the differentiation of exogenous seed cells into NB in periodontal fenestration defects.

#### Micro-CT analysis

**Figure 13:**
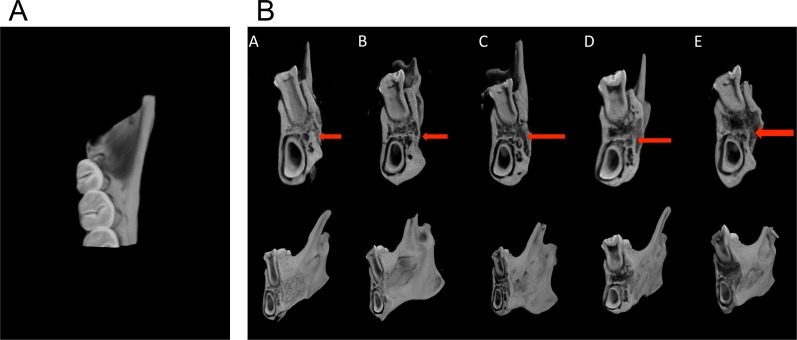
Micro-CT analysis of the repair of periodontal tissue defects in different groups **(A)** Diagrammatic sketch of the distal cross-section of the rat's first molar; **(B)** 3D reconstruction images of the distal cross-section of the first molars in different groups; red arrow indicates periodontal defects prepared for the surgery.

Figure 13A shows a diagrammatic sketch of the distal cross-section of the first molar selected from the mesiodistal angle on the mandible; sections taken in each group were consistent. Figure 13B shows a 3D reconstruction image of the distal cross-section of the first molar in different groups. Various degrees of bone repair were observed in the periodontal defects for each group. New bone density was different in each group, and the density of new bone tissues in the LentiV-shPHD2-MSC+Bio-Gide collagen membrane group was significantly higher than that in the other four groups.

Fifty consecutive sections were selected from the mesiodistal part of the rat mandible, especially from the distal root of the first molar to the entire root areas, as measurement areas. Quantitative analysis of osteogenesis-related parameters in the measurement area showed that the mean bone mineral density (BMD) in the LentiV-shPHD2-MSC+Bio-Gide collagen membrane group was significantly higher than that in the other four groups (P<0.05). The measured tissue volumes (TV) showed no significant differences between groups. Bone volume (BV) in the LentiV-shPHD2-MSC+Bio-Gide collagen membrane group was significantly higher than that in the other four groups (P<0.05). BV/TV ratios were different between groups, and the BV/TV ratio in the LentiV-shPHD2-MSC+Bio-Gide collagen membrane group was significantly higher than that in the other four groups (P<0.05) (Table 1).

**Table 1:** Related parameters of new bone tissues in the periodontal defect regions in the different groups *, #, Δ, *vs*. LentiV-shPHD2-MSC+Bio-Gide collagen membrane group, *P*<0.05

GROUP	Bone Volum(BV) (Mean±SD, mm^3^)	Tissue Volume(TV) (Mean±SD, mm^3^)	BV/TV (Mean±SD,%)	Bone Mineral Density (BMD) (Mean±SD, g/cm^3^)
LentiV-shPHD2- MSC+ Bio-Gide collagen membrane	0.6982±0.0589	1.4410±0.2893	48.69±4.69	0.6699±0.0739
LentiV-GFP-MSC+ Bio-Gide collagen membrane	0.5335±0.0332*	1.4280±0.2042	37.20±3.55#	0.5964±0.0514Δ
CON-MSC+Bio-Gide collagen membrane	0.5284±0.0074*	1.4684±0.1190	35.86±2.33#	0.4987±0.0847Δ
Simple Bio-Gide collagen membrane	0.4490±0.0669*	1.4051±0.1068	36.56±2.93#	0.4638±0.0754Δ
Blank control group	0.4199±0.0727*	1.4120±0.0408	25.16±4.08#	0.4134±0.0687Δ

## DISCUSSION

In recent years, tissue engineering technology and materials science have developed rapidly in the field of periodontal tissue regeneration. A new research trend in this field is the implantation of externally proliferated seed cells on collagen membranes for 3D structures and the construction of tissue-engineered compound for repairing periodontal tissue defects [21]. Several studies have used periodontal ligament cells (PDLCs) as seed cells to repair periodontal tissue defects [22]. Zhang J et al. demonstrated that, during osteogenic induction in an inflammatory microenvironment, bone marrow mesenchymal stem cells (BMMSCs) have stronger mineralization capabilities than PDLSCs, and increased expression levels of osteogenesis related genes [23]. Du J et al. confirmed that local injections of BMMSCs can repair periodontal defects caused by periodontitis in rats and inhibit the secretion of inflammatory factors in periodontal defects [24]. The above results indicate that BMMSCs may be a more ideal choice for repairing periodontal tissue defects caused by periodontitis. Bio-Gide membrane is a commercial product extensively applied in periodontal therapy. It is a collagen membrane with a 3D network structure that is primarily composed of collagen fibers that are biocompatible and biodegradable. The fibers can induce barrier membrane effects in periodontal tissues during regeneration [25, 26].

Sprague Dawley (SD)rats have similar periodontal tissue structure and function to humans; hence, rats were chosen to develop the periodontal defect model. Chronic defects induced by plaque and dental calculus, etc., are the root cause of periodontitis. However, considering that a longer period is needed to establish the effect of multiple factors, the method by King GN et al. [27] was selected to develop the rat periodontal fenestration defect model via surgical methods. In general, tissue-engineered compounds constructed using SD rat BMMSC Bio-Gide membranes were selected as experimental carriers to promote SD rat periodontal fenestration defect repair.

Many growth factors and relevant cytokines produce regulating effects during periodontal tissue regeneration. In past studies, was confirmed that the direct application of exogenous recombinant growth factors during tissue regeneration is ideal for periodontal tissue repair. For example, platelet-derived growth factor, insulin-like growth factor, fibroblast growth factor and bone morphogenetic protein have been used to treat extensive periodontal tissue defects in animal experiments and clinical practice and have been applied in bone incremental treatment around an implant [3]. However, different types of exogenous recombinant growth factors have significantly different validity periods based on their respective half-lives and cannot guarantee consistent benefits [28]. A lentiviral vector was selected in this study, which is a popular choice for an extensive range of cell infections. Lentiviral vectors can integrate exogenous genes into the target cell genome for persistent expression or silencing, thus promoting the continuous expression of downstream growth factors. The results of this study indicate that, after BMMSCs have been infected by a lentivirus RNA interference vector for 72 hours, Prolyl hydroxylase domain protein 2 (PHD2) can be effectively silenced at the gene and protein levels.

Prolyl hydroxylase domain protein 2 (PHD2) gene silencing can activate the hypoxia response pathway mediated by hypoxia-inducing factor (HIF) under normoxic conditions and promote the secretion of several vascular growth factors regulated by HIF-1α, e.g., VEGF, EPO, INOS and bFGF, etc.[14, 29, 34, 35]. vascular endothelial growth factor (VEGF)can promote the formation of new blood vessel networks in the pericementum, provide blood supply to fibroblasts in the pericementum, and create good conditions for periodontal tissue regeneration [30, 31]. Basic fibroblast growth factor(bFGF), an alkaline polypeptide that has been extensively studied, has chemotactic properties in endothelia; it can stimulate endothelia migration and proliferation and form new blood vessels [32, 33]. bFGF is an important mitogenic factor, an inducing factor for morphogenesis and differentiation, and plays an important role in promoting tissue repair and trauma recovery [34, 35]. Murakami et al. utilized recombinant basic human fibroblast growth factor to treat grade II furcation involvement in beagles, and the results indicated that periodontal defects were repaired [36]. When BMMSCs were infected by a lentiviral RNA interference vector for 72 hours to 1 week in this study, the secretion levels of VEGF and bFGF consistently increased compared to the control group. VEGF and bFGF levels also increased during osteogenic induction of infected cells but decreased only in the late phase of osteogenic induction due to decreased cell secretion. A tissue-engineered compound was constructed using BMMSC compound Bio-Gide collagen membranes after PHD2 gene silencing and was subcutaneously implanted into nude rats for 5 weeks. Greater local microvascular regeneration was observed than in control group. The above results suggest that lentiviral vector-mediated RNA interference can induce persistent and stable silencing of PHD2 *in vitro*, thus improving the secretion of angiogenic factors and promoting local neovascularization.

Many studies in recent years have confirmed that, except for strong angiogenesis, VEGF can also promote bone regeneration [37, 38]. VEGF can promote endothelia proliferation, which produces cytokines including bone morphogenetic protein and promotes bone repair and regeneration. It can also promote differentiation and growth of osteoblasts and osteoclast and participate in osteogenesis [39]. Grellier et al. confirmed in a co-culture system of osteoblasts and human umbilical vein endothelia that, as a paracrine cytokine, VEGF participates in early angiogenesis and promotes osteogenic differentiation [40]. All these results suggest that VEGF is important in angiogenesis and bone regeneration. Runt-related transcription factor 2(Runx2) is a relevant osteoblast regulating factor and has a major regulating effect on the differentiation of osteoblasts. It is one of the early parameters of osteogenic differentiation of cells and is critical for bone formation [41]. It has been confirmed that Runx-2 is an important osteogenesis-related factor, and Hif-1α is a relevant angiogenic factor [42]. These effects can indirectly or directly stimulate VEGF gene expression in mesenchymal stem cells during osteogenic differentiation, suggesting that Runx2 has certain vascularization effects during osteogenic differentiation [43]. Sun Hee Lee et al. demonstrated that Runx-2 can block the reactions between pVHL and HIF-1α by binding the proline residue (ODDD) of HIF-1α, thus stabilizing HIF-1α expression and stimulating vascularization in hypertrophic cartilage [44]. Another study also demonstrated that Bone morphogenetic protein(BMP2)combined with HIF-1α promoted osteogenic differentiation of mesenchymal stem cells [45]. In addition, several studies have confirmed that increased HIF-1α levels can improve alkaline phosphatase (ALP) expression levels and osteogenic differentiation-related parameters [46]. The results of this study indicate that, on days 4 and 7 of early osteogenic induction *in vitro* in BMMSCs after PHD2 gene silencing, the expression levels of Runx-2, ALP, Collagen Type I(COL-1)and osteocalcin (OCN)genes and proteins were higher than in the control group. The level of BMP2 secretion in early *in vitro* osteogenic induction was not significantly different from that in the control group, but its level persistently increased in the mid- and late phases of osteogenic induction and was significantly higher than in the control group. Alkaline phosphatase staining on days 7 and 14 of osteogenic induction and alizarin red staining on day 21 of osteogenic induction showed that, after PHD2 gene silencing, BMMSCs had higher osteogenic differentiation levels compared to the control group. Combined with studies performed by other researchers, under normoxic conditions, lentiviral RNA interference vector-mediated PHD2 gene silencing can promote osteogenic differentiation of BMMSCs *in vitro*, and mutually synergistic effects were observed among the expression levels of HIF-1α, osteogenic-related regulation factor (Runx-2, etc.) and angiogenic factors (VEGF, etc.).

Results from the Micro-CT 3D reconstruction and the related bone parameter analysis, as well as histological findings, demonstrated that, compared to the tissue-engineered compound in the control groups, the tissue-engineered compound constructed by combining BMMSCs after PHD2 gene silencing and Bio-Gide collagen membrane promoted better rat periodontal tissue repair, including NB, NC and NP. The effect of the NP repair could be caused by VEGF, bFGF, etc, which are favorable to the differentiation of fibroblast. Some researchers have applied compounds that can inhibit the human PHD2 gene in mice and found increased VEGF expression and new blood vessels [47]. Rios et al. used seed cells with RNA interference PHD2 gene in a goat bone defect model, and good bone regeneration was observed [48]. These results are consistent with the results of this study. As the HIF pathway can be activated under hypoxic conditions, which facilitates the accumulation of HIF-1α, it is necessary to include a hypoxic cultivation group as a positive control group in further studies. This would further confirm the results obtained by inhibiting the PHD2 gene under normoxic conditions found in this study.

Hydrogen peroxide was selected in this study at low concentrations as the reactive oxygen species (ROS) to imitate the stimulating effects of active oxygen BMMSCs under oxidative stress *in vitro*. Caspase-3 plays a very important mediating effect in cell apoptosis and is the most important final shear enzyme during cell apoptosis [49, 50]. The results of this study demonstrated that, after normally cultured BMMSCs were pretreated with supernatant collected from BMMSCs infected with the lentivirus RNA interference vector, cell apoptosis was decreased under oxidative stress *in vitro*. The expression levels of cleaved caspase-3 in BMMSCs also decreased under the same state. In this study, the conditional medium (CM) mentioned above exerted protective effects on the BMMSCs during oxidative stress, suggesting that the increased concentrations of vascular factors such as VEGF in the supernatant can be used as pro-survival factors to exert protective effects on tissue cells and to inhibit cell apoptosis to some extent [51, 52]. The results of this study also demonstrated that, after PHD2 gene silencing, BMMSCs also exhibited reduced apoptosis and expression levels of cleaved caspase-3 protein during oxidative stress, suggesting that PHD2 gene silencing can postpone apoptosis of BMMSCs during oxidative stress. Several studies have now shown that HIF-1α can inhibit cell apoptosis induced by hypoxia conditions, which suggests a self-protection mechanism of the cellular hypoxia response during the early stage. Bcl-xl is a member of the Bcl-2 family, and it also functions by inhibiting cell apoptosis and autophagy. It has been demonstrated that HIF-1α plays a direct regulatory role by interacting with the HRE element in the Bcl-xl promoter and that the anti-apoptotic effect of Bcl-xl is stronger than that of Bcl-2 [53]. Chen N et al. demonstrated that PHD2 gene inhibition can increase HIF-1α and activate anti-apoptosis gene Bcl-xl expression, thus inhibiting cell apoptosis and autophagy [54]. Takeda K et al. confirmed that the PHD2 inhibitor DMOG may activate the NF-κB pathway and produce anti-inflammatory effects [55]. Natarajan et al. found that, in *in vitro* tissue culture, PHD2 gene inhibition can reduce the expression of inflammatory and chemotactic factors by activating the HIF pathway and confer protective effects on myocardial cells to help them resist ischemic reperfusion [56]. The conclusions from the above reported studies may be helpful in explaining the specific mechanism of PHD2 gene silencing and how it enhances BMMSC resistance to oxidative stress. However, more evidence is required from further experimental studies.

Notably, in this study, PHD2 gene silencing delayed the proliferation rate of BMMSCs (within 1 week). In addition, lentiviral vector infection inhibited the early proliferation of cells, although the specific reasons require further studies. Additional studies are required to provide more evidence to support the longer duration of repair in regenerative therapies of periodontal tissues due to the effects of PHD2 gene silencing. Moreover, SD rats exhibit individual variations in their development and body shapes, and surgical procedures may also result in artificial errors, all of which may have influenced the experimental result analysis. Hence, further studies are required in appropriate animal models that have structural similarities to the human body to obtain more precise results.

## MATERIALS AND METHODS

### Cultivation of BMMSCs

Bone marrow mesenchymal stem cell (BMMSCs) of SD rats were purchased from AllCells, LLC (Alameda, CA, USA), isolated and purified, and their phenotype was identified. The cells were then passaged and proliferated to the third generation for later use. The cells were recovered and cultivated in an incubator at 37°C and 5% CO_2_. An exclusive mesenchymal stem cell medium (MSCM) from ScienCell Inc. (Carlsbad, CA, USA) containing low glucose DMEM, 5% FBS, 1% double antibody and 1% growth cytokines for BMMSCs was used.

### Design and construction of lentivirus RNA interference vector

Four pairs of short hairpin RNA (shRNA)interference sequences (Table 2) were designed for rat PHD2 (accession No: NM_178334) to construct recombinant shuttle plasmids and packaging plasmids (pGag/Pol, pRev, pVSV-G). Sequenced plasmids and transfection reagent RNA-mate were used to transfect 293 T cells. The supernatants with lentivirus particles were collected, which were then concentrated and purified to obtain lentivirus concentrate for subsequent experiments. Design of the shRNA interference sequence, construction and sequencing of plasmids, and packaging and purification of lentiviral vectors were performed by GenePharma Ltd. (Shanghai, China). All lentiviral vectors carried the green florescent protein (GFP) label. Negative controls of the lentiviral vector were also constructed and used in the negative control group. This vector carried no other exogenous gene except for the GFP label.

**Table 2:** Interference sequences for four pairs of shRNA designed for target genes

shRNA		5′- 3′
shPHD2-1	sense	GATCCGAACCCAAGTTTGATAGATTGTTCAAGAGACAATCTATCAAACTTGGGTTCTTTTTTG
	antisense	AATTCAAAAAAGAACCCAAGTTTGATAGATTGTCTCTTGAACAATCTATCAAACTTGGGTTCG
shPHD2-2	sense	GATCCGTCACGTCGATAACCCAAATGTTCAAGAGACATTTGGGTTATCGACGTGACTTTTTTG
	antisense	AATTCAAAAAAGTCACGTCGATAACCCAAATGTCTCTTGAACATTTGGGTTATCGACGTGACG
shPHD2-3	sense	GATCCGCATGAACAAGCACGGCATCTTTCAAGAGAAGATGCCGTGCTTGTTCATGCTTTTTTG
	antisense	AATTCAAAAAAGCATGAACAAGCACGGCATCTTCTCTTGAAAGATGCCGTGCTTGTTCATGCG
shPHD2-4	sense	GATCCGTGACTCTTCCAAGGACATCCTTCAAGAGAGGATGTCCTTGGAAGAGTCACTTTTTTG
	antisense	AATTCAAAAAAGTGACTCTTCCAAGGACATCCTCTCTTGAAGGATGTCCTTGGAAGAGTCACG

### Determining multiplicity of infection (MOI) for lentiviral vector in BMMSCs

Third-generation BMMSCs were inoculated into a 6-well plate at density of 2*10^5 cells/well and cultivated at 37°C and 5% CO_2_ in an incubator. Twenty-four hours after inoculation, a negative control lentiviral vector was added to all 4 wells to attain MOI values of 0 (control group), 100, 150 and 200, according to the respective cell count in each well. Supernatant cell fluid containing the virus was discarded 24 hours after infection, and fresh MSCM was added. The cells were then cultivated for another 48 hours at 37°C and 5% CO_2_. Routine optical microscopy and inverted fluorescence microscopy were used to observe cell infection in different groups and their respective statuses. Cells were digested and collected with 0.25% trypsin-EDTA (Gibco, Grand Island, NY, USA) and washed twice, and a BD FACSCalibur flow cytometer was used to analyze single-labeled fluorescence. The experiment was repeated 3 times, and data were analyzed by using FlowJo flow cytometry analysis software. The expression rate of GFP (FITC channel) in cells was used as an effective measure of the infection rate of the lentivirus in BMMSCs. The effective MOI that achieved the maximal infection rate was selected as the standard for lentiviral vectors to infect cells for all subsequent experiments.

### Identification of PHD2 gene to silence the lentiviral vector

Third-generation BMMSCs were inoculated in a 6-well plate at a density of 2*10^5 cells/well and cultivated at 37°C and 5% CO_2_ in an incubator. Twenty-four hours after inoculation, the MOI defined in step 3 was used as a reference to add concentrated lentiviral vectors (LentiV-shPHD2-1, LentiV-shPHD2-2, LentiV-shPHD2-3, LentiV-shPHD2-4) to each well. Virus concentrates with the lentiviral vector (LentiV-GFP) were added to the negative control group, while no virus solution was added to the blank control group. The cells were then cultivated. After 24 hours of inducing infection, the supernatant containing the lentivirus was discarded. Cells were cultivated for another 48 hours and then washed. The culture fluid was then discarded. Trizol lysate was used to lyse cells in each well and to extract the total RNA, and then SuperScript® VILO™ cDNA synthesis kit (Thermo Fisher Scientific, Waltham, MA, USA) was used for reverse transcription. TaqMan gene expression assay kits (Applied Biosystems, Grand Island, NY) were used to perform real-time quantitative PCR. PHD2 mRNA expression levels in different groups were quantitatively compared by using the ΔΔT method and expressed as 2^−ΔΔT^. RIPA lysate (Beyotime, China) was used to lyse cells in each well and to extract total protein for Western blot, with anti-PHD2-rabbit-mAb (Cell Signaling Technology, Danvers, MA, USA) and anti-HIF-1α-mouse-mAb (Abcam, Cambridge, MA, USA) as the primary antibodies and β-actin expression serving as an internal reference. Gray level analysis was performed using a Gel-Pro Analyzer. The above experiments were repeated 3 times. The results of gene and protein detection were used to screen out the effective PHD2 gene lentiviral RNA interference vector (LentiV-shPHD2) for subsequent experiments.

### *In vitro* study of biological behavior of BMMSCs after PHD2 gene silencing

Third generation BMMSCs were inoculated into a 6-well plate at a density of 2*10^5 cells/well and cultivated with MSCM in an incubator at 37°C and 5% CO_2_. The wells were divided into the following 3 groups 24 hours after inoculation: the LentiV-shPHD2-MSC group (lentiviral RNA interference vector), the LentiV-GFP-MSC group (negative control of lentiviral vector) and the CON-MSC group (no lentiviral vector). Fresh MSCM was added to continue the cultivation after 24 hours. The medium was then replaced every other day.

The supernatant of the cell culture was collected after 72 hours and after 1 week (day 7) from the different groups after infection with viral vector. A Quantikine Elisa kit (R&D Systems, Minneapolis, MN, USA) was used to detect concentrations of vascular endothelial growth factor (VEGF), basic fibroblast growth factor (bFGF) and bone morphogenic protein2(BMP2) in the supernatant. Three identical wells were used at different time points in each group. The supernatant was retained and stored at −20°C 72 hours after infection.

Seventy-two hours after infection, 0.25% trypsin-EDTA (Gibco, Grand Island, NY, USA) was used to digest and collect cells, which were further inoculated into a 96-well plate at a density of 4000 cells/well. Fresh MSCM was added every other day. On days 1, 3, 5 and 7 after inoculation, a cell counting kit-8 (Dojindo, Kumamoto, Japan) was introduced to each well, and the cells were incubated for another 3 hours. A microplate reader was used to read the absorbance values of different wells at a wavelength of 450 nm to compare cell viability at different time points between groups and to ultimately draw a cell proliferation curve after one week.

Seventy-two hours after infection, 0.25% trypsin-EDTA was used to digest and collect cells from each well of the 6-well plate. The cells were washed twice, fixed with 70% ethanol and stained in groups with PI/RNase staining buffer (BD Pharmingen, San Diego, CA, USA). Flow cytometry was used to detect cell cycles in different groups, and the data were analyzed by using a FlowJo flow cytometry analyzer.

Cells in the 6-well plate in different groups were observed 72 hours after infection. Wells with good cell growth and 80% to 90% cell fusion were selected, and the osteogenic induction medium was changed with the following ingredients: α-minimum essential medium (MEM) (Gibco, Grand Island, NY, USA), 10% fetal bovine serum (HyClone, South Logan, UT, USA), 1% penicillin-streptomycin solution (containing 10000 U/mL and 10000 μg/mL penicillin-streptomycin) (HyClone, South Logan, UT, USA), 10^−7^ mol/L dexamethasone (Sigma-Aldrich, St. Louis, MO, USA), 50 mg/L vitamin C (Sigma-Aldrich, St. Louis, MO, USA) and 10 mM β-glycerophosphate disodium (Sigma-Aldrich, St. Louis, MO, USA). The liquid was changed every 3 days. On days 4, 7, 10, 12, 14 and 21 during osteogenic induction, the supernatants were collected from cells in different groups. A Quantikine Elisa kit (R&D Systems, Minneapolis, MN, USA) was used to detect the concentrations of VEGF, bFGF and BMP2 in the collected supernatants. Three identical wells were used for each group at different time points. During osteogenic induction detection, the results were used to draw line charts depicting the trends in growth factor concentration changes in the supernatant for each group.

On day 4 and 7 during osteogenic induction, the culture medium was discarded from the 6-well plate, and the cells were then washed twice. Trizol lysate was used to lyse cells in each well and to extract total RNA. A SuperScript® VILO™ cDNA synthesis kit (Thermo Fisher Scientific, Waltham, MA, USA) was used for reverse transcription, and TaqMan gene expression assay kits (Applied Biosystems, Grand Island, NY) were used for real-time quantitative PCR. On days 4 and 7 during osteogenic induction, the mRNA levels in different groups were detected for osteogenic parameters including Runt-related transcription factor 2 (Runx-2), alkaline phosphatase (ALP), osteocalcin (OCN) and Collagen Type I (COL-1), quantitatively compared using the ΔΔT method, and expressed in 2^−ΔΔT^. PIPA lysate (Beyotime, China) was used to lyse cells in each well and to extract total proteins. On days 4 and 7 during osteogenic induction, Western blot was used to detect the expression levels of total proteins for osteogenic parameters including Runx-2, ALP, OCN and COL-1 with anti-Runx-2-rabbit-mAb (Cell Signaling Technology, Danvers, MA, USA), anti-alkaline phosphatase-rabbit-pAb (Abcam, Cambridge, MA, USA), anti-OCN-mouse-mAb (Arigo Biolaboratories, Hsinchu City, Taiwan) and anti-COL-1 mouse-mAb (Abcam, Cambridge, MA, USA) as the primary antibodies and β-actin serving as an internal reference. A Gel-Pro Analyzer was used to analyze the gray level. The above-mentioned experiments were repeated thrice.

On days 7 and 14 of osteogenic differentiation, a BCIP/NBT alkaline phosphatase color development kit (Beyotime, China) was used to perform alkaline phosphatase staining for cells in different groups. On day 21 of osteogenic differentiation, alizarin red S (Sigma-Aldrich, St. Louis, MO, USA) solution was used for staining mineralized nodules in cells of different groups.

### Role of PHD2 gene in enhancing BMMSC resistance to oxidative stress

Third generation BMMSCs were inoculated in a 96-well plate at a density of 10000 cells/well. All wells were divided into 5 groups with 5 identical wells set in each group. Cells were cultivated with MSCM at 37°C and 5% CO_2_. Twenty-four hours after inoculation, Opti-MEM I (1×) reduced serum media (GIBCO, Grand Island, NY, USA) was used to dilute 3% H_2_O_2_ (Sigma-Aldrich, St. Louis, MO, USA) into 5 concentration gradients (0 μM, 200 μM, 400 μM, 600 μM and 800 μM), which were used to replace the original medium and added to the wells. Cells were cultivated at 37°C and 5% CO_2_ for 3 and 6 hours, respectively. The culture medium with H_2_O_2_ was then discarded and replaced with fresh MSCM. A CCK-8 test kit was added to incubate the cells for another 3 hours. A microplate reader was then used to read the absorbance values in different wells at a wavelength of 450 nm to compare cell activities between different groups. The absorbance value in the cell wells containing 0 μM H_2_O_2_ was used as control. The H_2_O_2_ concentration that could markedly reduce cell activity was screened as the precondition to imitate oxidative stress *in vitro*.

Third generation BMMSCs were inoculated into a 12-well plate at a density of 1*10^5 cells/well. Cells were cultivated with MSCM at 37°C and 5% CO_2_ and cultivated for 48 hours. When at least 80% to 90% of the cells were fused, the wells were divided into the following 4 groups: (1) In the CON-MSC group, the original culture medium was retained. (2) In the LentiV-shPHD2-MSC-CM group, the original culture medium was discarded, and cell culture supernatant of the LentiV-shPHD2-MSC group was collected 72 hours after infection and used as the conditional medium (CM). The cells were then cultivated for another 6 hours at 37°C and 5% CO_2_. (3) In the LentiV-GFP-MSC-CM group, the original culture medium was discarded, and the cell culture supernatant in the LentiV-GFP-MSC group was collected 72 hours after infection and used as the CM. The cells were then cultivated for another 6 hours at 37°C and 5% CO_2_. (4) In the MSC group, the procedure was the same as in group (1) with the original culture medium retained. After cells in all groups were appropriately treated, the culture media in the latter three groups [(2), (3) and (4)] were changed to OPTI-MEM with selected H_2_O_2_ concentrations. Cells were further cultivated for 6 hours at 37°C and 5% CO_2_. After 6 hours, the culture medium with H_2_O_2_ was removed, and the cells from different groups were washed. RIPA lysate (Beyotime, China) was used to lyse cells for total proteins, while Western blot was used to detect expression levels of total proteins, including cleaved caspase-3 and total caspase-3. Anti-cleaved caspase-3/caspase-3-rabbit-mAb (Cell Signaling Technology, Danvers, MA, USA) was used as the first antibody, expression level for total caspase-3 and β-actin was used as the internal reference. A Gel-Pro Analyzer was used to analyze gray levels. All the above experiments were repeated thrice.

After 72 hours of exposure to infection, the cells were digested and collected from the LentiV-shPHD2-MSC group, the LentiV-GFP-MSC group and the CON-MSC groups, respectively, and inoculated into a 12-well plate at a density of 1*10^5 cells/well. The cells were then cultivated with MSCM at 37°C and 5% CO_2_. After 48 hours of inoculation, when 80% to 90% of the cells were fused, the original culture medium was changed to OPTI-MEM with a selected concentration of H_2_O_2_. The cells were then cultivated for another 6 hours at 37°C and 5% CO_2_. The culture medium with H_2_O_2_ was discarded after 6 hours, and the cells from different groups were then washed. RIPA lysate was used to lyse cells for total protein, while Western blot was used to detect the expression levels of total protein, including cleaved caspase-3 and total caspase-3. Anti-cleaved caspase-3/caspase-3-rabbit-mAb (Cell Signaling Technology Danvers, MA, USA) were used as the primary antibodies to detect the expression levels of total caspase-3, and β-actin was used as an internal reference. A Gel-Pro Analyzer was used to analyze gray levels. All the above experiments were repeated thrice.

Cell slides 20 mm in diameter were placed into the wells of a 12-well plate. Cells were collected and inoculated onto the cell slides as described in sections 6.2 and 6.3 and appropriately treated. After cultivating for 48 hours, when 80% to 90% of the cells were fused, the original culture medium was changed to OPTI-MEM with a selected concentration of H_2_O_2_. The cells were then cultivated for another 3 hours at 37°C and 5% CO_2_. Three hours later, the culture medium with H_2_O_2_ was removed, and cells on cell slides of each group were washed and then fixed. *In situ* cell death detection kit (TMR red) (Roche, Basel, Switzerland) and DAPI staining reagent (Beyotime, China) were used for TUNEL staining and DAPI staining. Fluorescence confocal microscopy images were collected. Six different visual fields were selected for each group of cells to count cells with positive TUNEL staining. The percentage of cells with positive TUNEL staining (count of cells with positive TUNEL staining/count of cells with positive DAPI staining) were compared and analyzed between different groups.

After cells were treated as described in section 6.2, the culture medium was changed to OPTI-MEM with a selected concentration of H_2_O_2_. The cells were then cultivated for another 6 hours at 37°C and 5% CO_2_. Six hours later, the culture medium with H_2_O_2_ was removed. Cells in different groups were digested and collected and then stained using an FITC Annexin V/PI apoptosis kit (BD Pharmingen, San Diego, CA, USA). Cells were then treated as described in section 6.3, and the culture medium was changed to OPTI-MEM with a selected concentration of H_2_O_2_. The cells were cultivated for another 6 hours at 37°C and 5% CO_2_. Cells in each group were digested and collected and then stained using an APC Annexin V/7-AAD apoptosis kit (BD Pharmingen, San Diego, CA, USA). Flow cytometry was used to inject samples, to analyze the stained cells, and to compare the apoptosis rate between groups (apoptosis rate = early apoptosis rate + late apoptosis rate). The above experiments were repeated thrice.

### Construction of tissue-engineered compound and observation by scanning electron microscopy

Under sterile conditions, a Bio-Gide collagen membrane (25×25 mm, Geistlich, Switzerland) was cut into 5×2 mm pieces. Each piece was placed into the wells of a 24-well plate with the coarse side of the collagen membrane turned upward. Then, 0.25 trypsin-EDTA was used to digest and collect cells 72 hours after lentiviral infection, and then cells were re-suspended into MSCM and inoculated onto the coarse surface of the cut collagen membrane in 3 groups at a density of 1*10^4 cells/well (LentiV-shPHD2-MSC, LentiV-GFP-MSC and CON-MSC groups). Only MSCM without cells was added in the blank control group. Cells were then cultivated at 37°C and 5% CO_2_ to prepare a tissue-engineered compound. Seventy-two hours after inoculation, cell growth on the collagen membrane was observed in the different groups via fluorescence confocal microscopy. The culture medium was then removed from each well. The collagen membrane was washed and then fixed in 2.5% glutaraldehyde at 4°C for 3 hours. After fixation, 30%, 50%, 70%, 80%, 90% and 100% ethanol were for gradient dehydration at room temperature for 15 minutes at each concentration gradient. The membrane was air-dried overnight at room temperature after dehydration. Metal spraying was conducted for the air-dried collagen membrane the next day. An SU8200 scanning electron microscope (Hitachi, Japan) was used to observe the colonization of cells on the collagen membranes.

### Subcutaneous transplantation of the tissue-engineered compounds in nude rats

Seventy-two hours after cells were inoculated onto the collagen membrane, the constructed tissue-engineered compound was surgically implanted subcutaneously in the backs of 4-week-old female nude rats in 4 different groups (LentiV-shPHD2-MSC+Bio-Gide collagen membrane group, LentiV-GFP-MSC+Bio-Gide collagen membrane group, CON-MSC+Bio-Gide collagen membrane group, simple Bio-Gide collagen membrane group). The muscle layer was not injured during the procedure. Nude rats were provided by Nanjing University's Model Animal Research Center. The rats were euthanized 5 weeks after surgery. Samples were collected, fixed in 4% paraformaldehyde, dehydrated, embedded and sectioned, and stained using hematoxylin & eosin (HE), MASSON and CD31 (Abcam, Cambridge, MA, USA)immuno histochemistry methods. Histological sections of samples from different groups were observed and analyzed.

### Rat periodontal fenestration defect model and tissue-engineered compound implantation

Eighty 4-week-old male SD rats (provided by Model Animal Research Center of Nanjing University) were divided into five groups of 16. Rats were fed and allowed to adapt to their conditions for one week prior to surgery. The five groups were as follows:

- LentiV-shPHD2-MSC+Bio-Gide collagen membrane
- LentiV-GFP-MSC+Bio-Gide collagen membrane
- CON-MSC+Bio-Gide collagen membrane
- Simple Bio-Gide collagen membrane
- Blank control group

**Figure 14:**
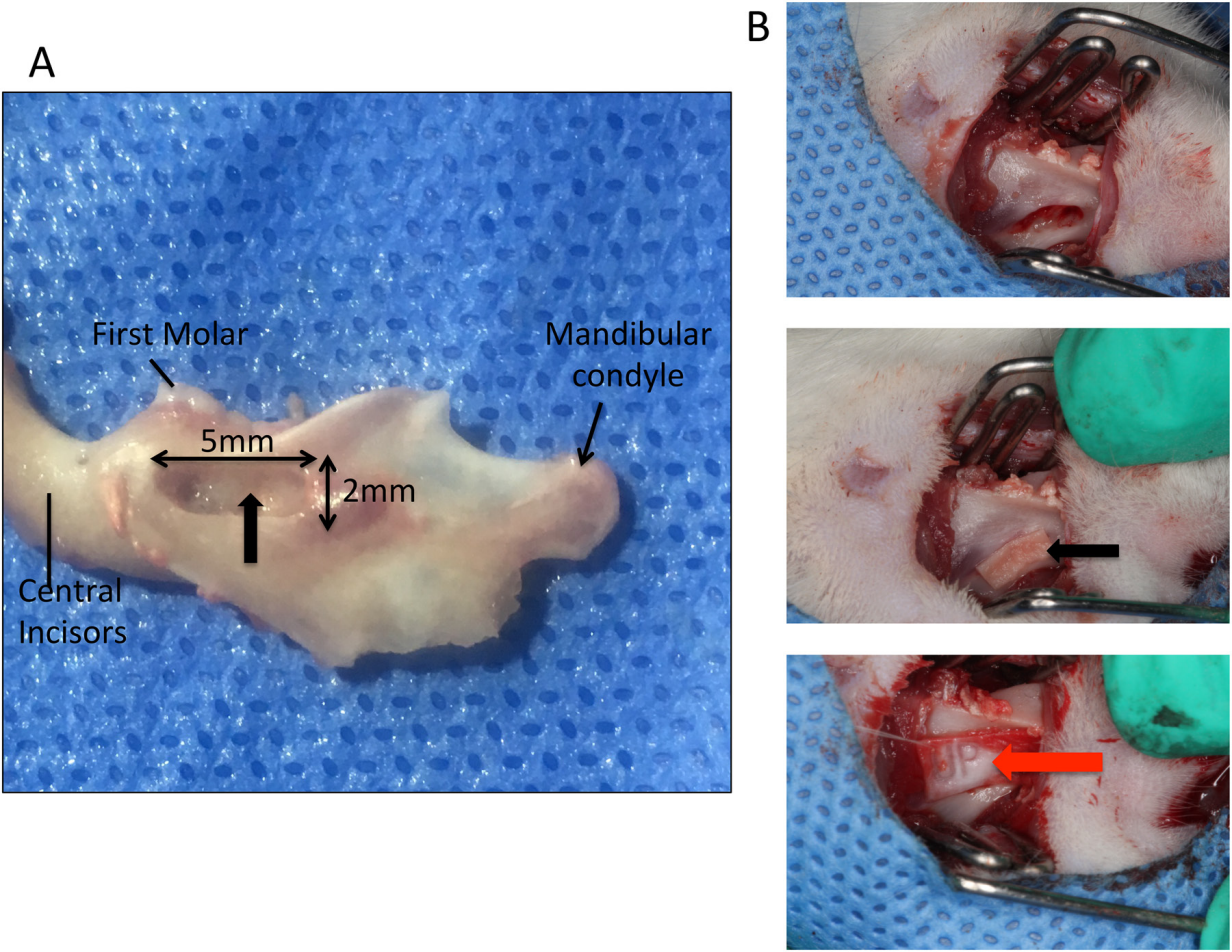
The diagrammatic sketch of the rat periodontal fenestration defect model **(A)** The diagrammatic sketch of the rat periodontal fenestration defect model, the black arrow indicates the defect location; **(B)** The diagrammatic sketch of the surgical course, the black arrow indicates the placement of the tissue-engineered compound, and the red arrow indicates the Bio-Gide barrier membrane covering the surface.

Rats were anesthetized intraperitoneally with 2% pentobarbital sodium (0.3 ml / 100 g) injection before surgery. The skin was routinely prepared for surgery and sterilized. A 2-cm-long mesiodistal incision was made on the skin at the inferior margin of the left mandible, approximately 1 cm from the angle of the mouth. Tissues were separated in layers, the masseter muscle and periosteum were bluntly separated, and the whole layer was manipulated to expose the mandibular bone. At the corresponding roots of the first and second molars, a manual electric drill and a ball drill with a 1.5-mm-diameter tip were used to superficially grind the cortical bone at a low speed, up to 2 mm from the alveolar ridge, to create a 5-mm defect in the mesiodistal direction, 1.5 mm in the buccolingual direction and 2 mm in the crown-root direction (Figure 14). A periodontal probe was used to measure the defect's size, and a small, sharp curette was used to scrape the residual periodontal ligament, cementum and dentin surfaces to make sure that the defect reached blow the root surface. A periodontal defect model was constructed in the left mandible of each rat.

As per the groups assigned above, the compound was placed into the prepared periodontal defect with the cell surface downward directly facing defect site. No material was placed in the blank control group. However, a 6×3 mm Bio-Gide membrane was placed onto the periodontal defect surface as a barrier membrane with the membrane margin exceeding the bone defect margin by approximately 0.5 mm. The incision was closed and sewn in layers. Three days after surgery, penicillin potassium was intraperitoneally injected (400 000 U / animal) to prevent infection. Three and eight weeks after surgery, all rats were euthanized by overdose with anesthetics in two batches respectively (40 in each batch).

### Histological observation and immunofluorescence images

Three weeks after surgery, the left mandible (from the central incisors to the mandibular angle) was completely dissected from each rat and fixed in 4% paraformaldehyde for 48 hours and then flushed overnight with water. The mandible was trimmed to remove excess tissue mesiodistally and then placed into 10% EDTA decalcifying solution for 2 months. The decalcifying fluid was changed every 3 days until there was no resistance to pinprick. After decalcification, the specimen was flushed overnight with water, then dehydrated with a gradient series of 40%, 50%, 60%, 70%, 80%, 90% and 95% ethanol for 12 hours each, and finally soaked in 95% ethanol and xylene solution (1:1) for 12 hours to make the specimen transparent. The specimen was embedded from the crown to the root in a wax dip. The specimen was then sectioned and observed under a microscope. Buccal tissue defect was observed on the distal root of the first molar for each specimen. The specimens were consecutively sectioned to a thickness of 5 μm, and 3 sections were selected from each 50 μm. Histological sections were stained by using HE and MASSON methods. Periodontal tissue regeneration was observed under a microscope and photographed. DAPI staining reagent (Beyontime, China) and anti-tublin antibody (Abcam, Cambridge, MA, USA) were used to stain nuclei and cytoskeleton of the sections in the LentiV-shPHD2-MSC+Bio-Gide collagen membrane group, the LentiV-GFP-MSC+Bio-Gide collagen membrane group and the CON-MSC+Bio-Gide collagen membrane group. Fluorescence confocal microscopy images were collected to observe the morphology and GFP expression in new alveolar bone of the three groups.

### Micro-CT 3D reconstruction and rat mandible data analysis

Eight weeks after surgery, the rat's mandible was fixed for 48 hours, a SkyScan1176 micro-CT scanner (Bruker Corporation, Germany) was used to perform CT scanning, and data reconstruction of all samples at a thickness of 18 μm/seam under a source voltage of 65 kV/source current 385 μA. CTvox software was used for 3D model reconstruction of the data. From the mesiodistal cross-section of the rat mandible, 50 consecutive slices were selected from the distal root of the first molar as the measurement region. CTAn software was used to measure osteogenic parameters in this region (bone mineral density, BMD; tissue volume, TV; bone volume, BV; BV/TV). Sample size n=8 for each group.

### Statistical analysis

All datas above are presented as the means ± SD. SPSS18.0 statistical software was used. After testing for normality and homogeneity of variance (the homogeneity test level α=0.05), one-way ANOVA analysis was used to evaluate statistical significance of the differences between two groups. P < 0.05 was considered statistically significant.

### Ethical approval

All the animal procedures conforms with the Guide for the Care and Use of Laboratory Animals published by the US National Institutes of Health (NIH Publication No. 85-23, revised 1996). The study protocol was approved by the animal care and use committee of the Nanjing University.

## CONCLUSION

Prolyl hydroxylase domain 2 gene silencing in bone marrow mesenchymal stem cells under normoxic conditions can promote periodontal tissue defect repair in SD rats and validate the resistance of cells to oxidative stress *in vitro*.
